# Quantitative Proteomics of Sleep-Deprived Mouse Brains Reveals Global Changes in Mitochondrial Proteins

**DOI:** 10.1371/journal.pone.0163500

**Published:** 2016-09-29

**Authors:** Jing Ren, Mei-Jun Zhang, Tie-Mei Li, Ju-en Zhang, Rui Lin, She Chen, Minmin Luo, Meng-Qiu Dong

**Affiliations:** National Institute of Biological Sciences, Beijing, China; Hopital du Sacre-Coeur de Montreal, CANADA

## Abstract

Sleep is a ubiquitous, tightly regulated, and evolutionarily conserved behavior observed in almost all animals. Prolonged sleep deprivation can be fatal, indicating that sleep is a physiological necessity. However, little is known about its core function. To gain insight into this mystery, we used advanced quantitative proteomics technology to survey the global changes in brain protein abundance. Aiming to gain a comprehensive profile, our proteomics workflow included filter-aided sample preparation (FASP), which increased the coverage of membrane proteins; tandem mass tag (TMT) labeling, for relative quantitation; and high resolution, high mass accuracy, high throughput mass spectrometry (MS). In total, we obtained the relative abundance ratios of 9888 proteins encoded by 6070 genes. Interestingly, we observed significant enrichment for mitochondrial proteins among the differentially expressed proteins. This finding suggests that sleep deprivation strongly affects signaling pathways that govern either energy metabolism or responses to mitochondrial stress. Additionally, the differentially-expressed proteins are enriched in pathways implicated in age-dependent neurodegenerative diseases, including Parkinson’s, Huntington’s, and Alzheimer’s, hinting at possible connections between sleep loss, mitochondrial stress, and neurodegeneration.

## Introduction

Almost all animals need to sleep, and prolonged wakefulness leads to overwhelming sleep pressure and neurocognitive defects, including reduced performance in sensory perception, motor action, memory, attention, and emotion [1]. Continuous sleep deprivation can even cause lethality in rats and drosophila [2–4]. Sleep is conserved from invertebrates to mammals, suggesting that it serves essential, fundamental functions in biochemical systems.

The molecular basis of sleep has been investigated repeatedly using genome-wide expression profiling methods. Using transcriptome analyses (mostly cDNA microarrays), more than ten research teams have compared the brains of rats, mice, sparrows, or flies when the animals were awake vs. when they were asleep. Many differentially-expressed transcripts were identified between these two states [5–18]. The results from these studies suggest that sleep and wakefulness have particularly impactful effects on cellular processes related to energy metabolism, synaptic potentiation, and responses to cellular stress [19,20]. However, it is known that mRNA abundance changes often do not correlate with protein abundance changes, owing to translational and post-translational regulation dynamics. It is proteins that carry out most cellular functions [21]. Therefore, a global survey of the protein abundance changes induced by sleep deprivation should in theory provide unique and meaningful biological insight into the fundamental need for sleep.

Several previous studies have monitored protein abundance changes after sleep deprivation. The first study was carried out by Basheer *et*. *al*. in 2005 [22], using two-dimensional (2D) gel electrophoresis coupled with mass spectrometry (MS) to quantify the proteomic changes in the basal forebrain of rat. This method was also employed by two other groups to analyze the proteomic changes in the mouse cortex and the rat hippocampus [23,24]. Limited by the analytical methodology, the coverage of the proteome in these studies was relatively low. Although up to 4000 protein spots were resolved on 2D gels, most of the spots showed no apparent abundance differences, and their identities were not determined; fewer than 43 proteins were found to be differentially expressed following sleep deprivation. In 2009, Cirelli *et al*. employed ProteinChip technology combined with surface-enhanced laser desorption-ionization and time-of-flight mass spectrometry to compare cerebral cortex proteins in rats that were either asleep or awake [25]. They identified 15 candidate biomarkers (eight for “sleep”, four for “waking”, and four for “long sleep deprivation”) from among 1055 protein peaks. Another recent study combined 1D and 2D gels and liquid chromatography (LC)-MS, and quantified changes in the abundance of 466 proteins in astrocytes of the rat hypothalamus after chronic partial sleep deprivation for seven days [26]. In recent years, MS-based proteomics has progressed tremendously in all aspects, including sample preparation, quantitation strategies, analytical hardware, and interpretive software[27]. Current proteomics technologies based on liquid chromatography coupled with tandem mass spectrometry (LC-MS/MS) can identify and quantify thousands of proteins, including proteins that are too large or too small and/or too acidic or too alkaline to be monitored with 2D gels [28]. For example, nearly 4000 proteins can be identified, and at a 1% false discovery rate (FDR), in a one-hour LC-MS/MS analysis [29]. In light of these advancements, it seemed likely that the use of cutting-edge quantitative proteomics technologies would dramatically increase the comprehensiveness of the coverage of the proteome of SD animal samples and would yield novel insights into the biology of sleep.

To quantify the relative protein abundance changes with a deep coverage of the proteome, we combined into our workflow filter-aided sample preparation (FASP) [30], stable isotope labeling, and high resolution, high mass accuracy, high throughput mass spectrometry. The FASP method features gel-free sample processing using ultrafiltration spin columns to facilitate sample cleanup and the digestion of proteins, especially of membrane proteins that had previously been a challenge for proteomics because of their poor solubility [31,32]. For accurate quantitation, we chose stable isotope labeling using a 2-plex tandem mass tag (2-plex TMT, *m/z* 126 *vs*. 127, one incorporated into control samples and the other into SD samples) and a fast-scanning, high-resolution orbitrap mass spectrometer to achieve precise protein measurements [33–36]. We quantified a total of 9888 proteins encoded by 6070 genes; this is the most comprehensive proteomic profile of SD mouse brain to date. By using two analysis pipelines in parallel to quantify the protein levels, we identified the differentially-expressed proteins induced by sleep deprivation. Mitochondrial proteins involved in energy metabolism and cellular stress were enriched among the differentially-expressed proteins Further analysis of these results also hinted at possible connections between sleep loss, mitochondrial stress, and neurodegeneration.

## Materials and Methods

### Animals

Animal care and use were carried out in accordance with the governmental policies of China, and the experimental protocols were approved by the Animal Care and Use Committee of the National Institute of Biological Sciences, Beijing. At the end of the sleep deprivation period, mice were sacrificed by cervical dislocation performed by a well-trained executor in the shortest possible time, and all efforts were made to minimize suffering of the animals used in this study.

Male C57BL/6N mice (aged 9–10 weeks, Vital River Lab Animals Co, Beijing) were housed under specific pathogen-free conditions at a constant room temperature of 24 ± 1°C with a 12 h:12 h light-dark cycle (Light on 9AM-9PM). A total of fifty-one mice were used for three experimental replications as follows: 18 mice in the first and third replications and 15 mice in the second replication. In each experimental replication, the mice were equally distributed among the control group, the gentle handling-induced sleep-deprived group (GSD), and the locomotion-induced sleep-deprived group (LSD) (*i*.*e*., n = 6 for the first and third replications; n = 5 for the second replication). Mice from the same control or experimental group were housed in the same cage, with nesting material and refuges provided.

### Sleep deprivation and tissue collection

We applied two procedures to induce SD. This experimental design decision was made with the aim of excluding particular response effects that may have resulted from some aspect of the treatment protocol itself, rather than from the sleep loss that the protocol achieved. Mice were allowed to habituate in individual lanes of a treadmill (Taimeng FT200, Chengdu, China) and given free access to food and water for 3 days (Light on 9 AM-9 PM). On the fourth day, the procedure commenced at 9 AM and lasted until 7 PM (10 hours). The mice in the LSD group were forced to walk on a treadmill with a continuous speed of 5 m/min. In this setup, if an animal stopped walking, it would find itself in a small chamber where it would receive mild electric foot shocks continuously until the animal resumed walking on the treadmill. Mice in the GSD group were kept awake for 10 hours by gentle handling, as described previously[15,37]. Briefly, at any moment from 9 AM through 7 PM, if no motor activity was observed, the animal’s fur was stroked with an artist’s brush by an experimenter. Control mice were placed on a non-moving treadmill for 10 hours at the same time. At 7 PM, all of the mice were sacrificed by cervical dislocation. The entire brain of each animal (excluding the olfactory bulb) was dissected immediately and snap frozen in liquid nitrogen. All tissue samples were stored at -80°C until use.

### Lysate preparation and protein digestion

The soluble and insoluble fractions were prepared by tissue homogenization and extraction as previously described, with minor modifications [38]. All procedures were carried out at 4°C unless otherwise specified. Each brain was homogenized in lysis buffer (2 M NaCl, 10 mM HEPES/NaOH pH 7.4, 1 mM EDTA, 1× Roche protease inhibitors) with 2 volumes of glass beads using a FastPrep-24 homogenizer (MP Biomedicals, Santa Ana, CA, USA) at 6.0 m/sec, 20 sec/pulse ×3 pulses. The homogenates (500 μl) were centrifuged in a benchtop centrifuge (HettichMikro 22R) at 30,000 ×*g* for 20 min, and the S1 supernatant was discarded. The pellets were re-homogenized in 1 ml of 0.1 M Na_2_CO_3_, 1 mM EDTA (pH 11.3) and incubated for 30 min before centrifugation. The S2 supernatant was saved. The pellets were extracted with 5 M urea, 100 mM NaCl, 10 mM HEPES (pH 7.4) and 1 mM EDTA and centrifuged at 30,000 ×*g* for 20 min. The S3 supernatant was again saved. The remaining pellets were washed twice with 0.1 M Tris/HCl pH 7.6 (14,000 ×*g* for 10 min), and the supernatant (W1 and W2) and pellet (insoluble fraction) were saved. Supernatants S2, S3, W1, and W2 were then combined; this combined sample was referred to as the soluble fraction, and the proteins from the soluble fraction were precipitated with methanol/chloroform. Pellets from both the soluble fraction and the insoluble fraction were solubilized in 0.1 ml 4% SDS, 0.1 M Tris/HCl (pH 7.6). The protein concentration was determined with a BCA Protein Assay Kit (Thermo).

Proteins were digested using the previously described FASP method [39] with 10K Nanosep Centrifugal Devices with an Omega Membrane (Pall Corporation, OD010C34). After alkylation and repetitive ultrafiltration, the concentrate was digested with trypsin (1:100) for 16 hours at 37°C. The peptides were collected by centrifugation, and the concentration was determined with a NanoDrop spectrophotometer (Thermo, ND1000) at 280 nm.

### Tandem Mass Tag (TMT) labeling and cation exchange-based fractionation of peptides

TMT reagents (Thermo scientific, 90065) were used to quantitatively label the peptides. For each biological replicate, 100 μg of peptides from the control brains were labeled with TMT-126, and 50 μg of peptides from the GSD or LSD brains were labeled with TMT-127. After the reactions were quenched, all 50 μg of the TMT-127-labeled peptides from the GSD or LSD groups were mixed with 50 μg of the TMT-126-labeled peptides from the control. Thus four sample groupings were generated from each of the experimental replications: CG-S (Control + GSD, Soluble), CL-S (Control + LSD, Soluble), CG-I (Control + GSD, Insoluble), CL-I (Control + LSD, Insoluble). The concentration of acetonitrile (ACN) in the combined samples was adjusted to 5% with ddH_2_O. The samples were stored at −80°C until use.

Strong cation exchange (SCX)-based off-line fractionation of peptides was performed as previously described, with modifications [40]. Briefly, 20 μg of labeled peptides were loaded onto a 250 μm (ID) two-phase column. This column contained a 2-cm-long reverse phase section (3 μm, 125 Å, Luna C18 resin from Phenomenex) upstream of a 2-cm-long SCX section (5 μm, 120 Å SCX resin from Whatman) and had a frit positioned at the end. After desalting with buffer A (5% ACN, 0.1% formic acid (FA), the peptides were eluted from the reverse phase column to the SCX resin with buffer B (80% ACN, 0.1% FA). Ten SCX fractions were collected, which were eluted with 5 μl of, respectively, 25, 50, 75, 100, 125, 150, 250, 500, 1,000, and 2,000 mM ammonium acetate. One-fifth of each eluate was analyzed via LC-MS/MS.

### Mass spectrometry (MS)

The peptides were loaded to a pre-column (ID: 75 μm; length: 8 cm) packed with 10–15 μm spherical C18 revered phase particles (YMC). The pre-column was connected with a piece of Teflon tubing to an analytical column (ID: 50 μm; length: 15 cm) packed with YMC 5 μm spherical C18 revered phase particles. The tip size of the analytical column was approximately 2 μm, and the flow rate was estimated to be 20–50 nl/min. To elute peptides from the column, an Agilent 1100 series binary pump system (Agilent) was used to generate the following HPLC gradient: a 120-min run with 90-min linear gradient from 100% buffer A (0.2 mM acetic acid in water) to 35% buffer B (70% ACN, 0.2 mM acetic acid), which then continued with a 15-min gradient from 35% to 100% buffer B.

The eluted peptides were sprayed directly into an LTQ-OrbitrapVelos mass spectrometer via a nano-ESI ion source. Full MS scans were acquired with a resolution of 30,000 at m/z 400 in the Orbitrap analyzer. The 10 most intense ions were fragmented by high-energy collisional dissociation (HCD) in the octopole collision cell. The spectra for the HCD fragment ions were acquired in the Orbitrap analyzer with a resolution of 7,500 at m/z 400.

### MS data analysis

We used two data analysis pipelines; one was Proteome Discoverer (PD, v.1.3.0. 339, Thermo Scientific) and the other was ProLuCID v.1.3.1 for peptide and protein identification [41] in conjunction with Census 1.57 for quantitation [42].

In the first pipeline, the raw files were searched against the IPI (International Protein Index) mouse database version 3.83 (EBI-IPI_mouse_3.83, release date May 28, 2011) using the search engine SEQUEST [43] embedded in PD. Peptides were identified by specifying trypsin as the protease, with no more than three missed cleavage sites, a precursor mass tolerance of 2,000 ppm, a fragment ion tolerance of 0.02 Da, and carbamidomethylation (C) and TMT-2plex (K, N-term) as fixed modifications. The quantification method was set as “TMT 2plex” with the mass tolerance of reporter ions at 20 ppm. The precursor mass tolerance was set to 2,000 ppm to allow for the monoisotopic and up to three isotopic peaks for database searching. The protein and peptide spectrum match lists were generated using a 1% false discovery rate (FDR) cutoff at the peptide level, and required a peptide mass accuracy of 20 ppm. The latter requirement was implemented using the following formula:

$$\frac{observed\;molecular\;value–theoretical\;molecular\;value–i*1.003355}{theoretical\;molecular\;value*0.000001}<20,\;\text{where}\;\text{i}=1,\;2,\;3,\;\text{or}\;4.$$

In the second pipeline, the MS/MS spectra were searched using ProLuCID against the same IPI database for peptide and protein identifications. The following parameters were specified for database searching: digestion enzyme: trypsin; precursor mass tolerance: 4550 ppm; fragment mass tolerance: 20 ppm; static amino acid modifications: carbamidomethylation of cysteine (57.021464 C) and TMT labeling of primary amino groups (229.1629 K, N-ter) [44]. The ProLuCID search results were filtered using DTASelect v. 2.0.25 [45] by requiring 1% FDR at the peptide level (-fp 0.01), 20 ppm precursor mass accuracy (-DM 20), and a minimum Z score of 4 (-S 4). Finally, Census software was used to extract the relative intensities of the reporter ions for each peptide from the identified tandem mass spectra [42], with an intensity threshold of 150.

An in-house Perl script was used to remove peptides whose MS2 spectra were suspected to contain a significant amount of interfering signals from co-eluting peptides of similar m/z values. If the sum of the interference peaks in the MS spectrum within the isolation window of the target peak (±1 m/z) was more than 50% of the sum of the target peak and its isotopic peaks($\frac{sum\;(interference\;peaks)}{sum\;(target\;peaks)}>0.5$), then the MS/MS spectrum of the target peptide was disqualified and excluded.

At least two peptides were required for the quantification of each protein. The protein identification and peptide quantification results from each of these two quantitative analyses (Proteome Discover and Census) were normalized using the median of the peptide ratios. After normalization, the two results were merged. The median ratio of a protein's subordinate peptides was used as the relative abundance ratio for that protein.

### Bioinformatics and statistics

The relative expression levels (RELs) of the proteins were corrected by setting the median of the log2 (REL) to 1 in each experiment. The reproducibility of the experiments was tested using Pearson correlation and Spearman correlation [46]. We used the LIMMA package in the R language for statistical computing [47] to examine the protein expression changes induced by SD treatment. The Benjamini-Yekutieli multi-test correction method [48] was used to control the false discovery rate (significance threshold = 0.05).

In order to minimize variations among the three experimental replications for each sleep deprivation method, the differentially expressed proteins of the paired control and GSD/LSD samples were determined based on the deviation of a given protein from the normalized interquartile range (NIQR) of all proteins within each replication [49]. In the CG-S, CL-S, CG-I, or CL-I sample groupings, a protein was recognized as being up-regulated if its relative abundance ratio (SD treatment/control) met any of the following conditions: (1) ≥1+3*NIQR in one of the three replicates and ≥1+1.5*NIQR in another, irrespective of the value in the third; (2) ≥1+2*NIQR in two replicates and ≥1+1.0*NIQR or NA in the remaining one; and (3) >1+1.5*NIQR in all three replicates. Criteria for determining the down-regulated proteins were based on equivalent reductions from the normalized value of 1. Proteins that presented paradoxical changes between the CG and CL groups were discarded without further investigation (S1 Table).

Gene Ontology (GO) enrichment analysis was assessed with the BINGO 2.44 program, including gene_association.mgi (Submission Date: 3/28/2013) and gene_ontology.obo (version 3/29/2013) [50]. The identified proteins were used as the background in the hypergeometric tests, and the results were corrected using a Benjamin & Hochberg method with a FDR cutoff of 0.05. The g:Profiler was applied to identify statistically-enriched human disease genes through searching the KEGG and Human Phenotype Ontology databases [51]. Venn plots, bar plots, and heat map plots were constructed with R [52].

### Western blot analysis

Whole brain homogenate was used for western blot quantification. Total protein samples were resolved by SDS-PAGE, transferred to PVDF membranes, and analyzed by western blotting. The antibodies used were Hspd1/Hsp60 (4870, Cell Signaling, 1:1000 dilution)) and Sod2 (ab13533, Abcam, 1:5000 dilution). We used GelQuantNET to measure the signal intensities of the protein bands. Three measurements were averaged and normalized to the total protein amount from the Coomassie staining signal intensity.

## Results

### Quantification of sleep-deprived mouse brain proteomes by combining FASP and TMT-labeling

**Fig 1:**
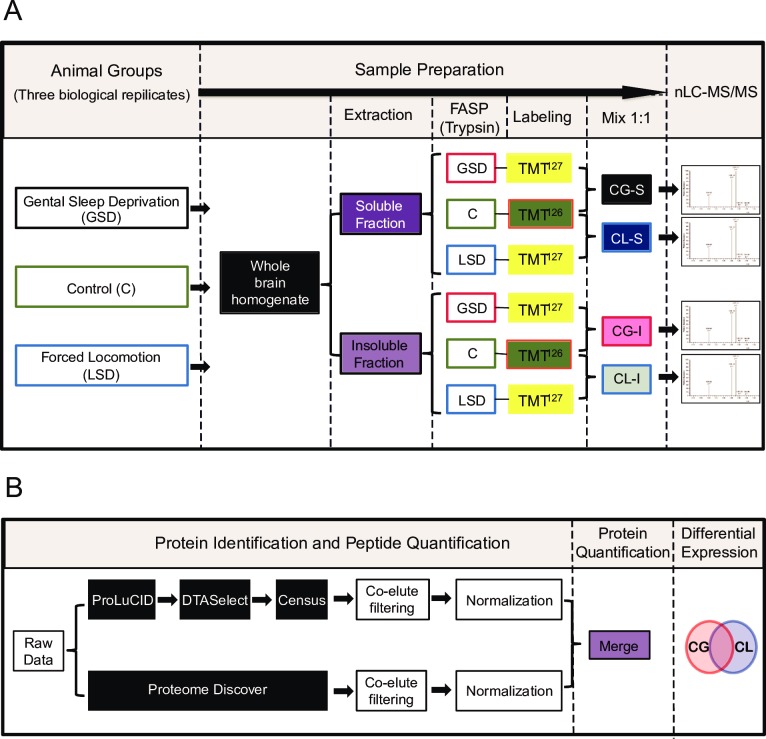
Workflow schematics. (**A**) Methods of sleep deprivation, sample preparation, and quantitative mass spectrometry analysis. Mice were deprived of sleep for 10 hours during their normal sleeping time by either gentle handling (GSD) or forced locomotion (LSD). Whole brains from sleep-deprived (SD) mice, as well as those from control mice, were homogenized, and the soluble and insoluble fractions were extracted and digested into peptides using the FASP method. TMT^126^-labeled peptides from control group and TMT^127^-labeled peptides from the GSD or LSD group were mixed at a 1:1 mass/mass ratio before off-line SCX fractionation and on-line reverse-phase LC-MS analysis on an LTQ OrbitrapVelos mass spectrometer. CG-S: combined soluble fractions of the control group and the GSD group (Control + GSD, Soluble); CL-S: combined soluble fractions of the control group and the LSD group (Control + LSD, Soluble); CG-I: combined insoluble fractions of the control group and the GSD group (Control + GSD, Insoluble); CL-I: combined insoluble fractions of the control group and the LSD group (Control + LSD, Insoluble). (**B**) Protein identification and quantification using two separate data analysis pipelines. The results were integrated; differentially expressed proteins and affected pathways were analyzed.

“Gentle handling”, “forced locomotion”, and the “punitive method” are three of the most commonly used total sleep deprivation methods, but each has known limitations and disadvantages [53][54]. The Gentle handling method may not fully preclude non-rapid eye movement sleep (NREM) [55,56]. The forced locomotion and punitive methods are known to affect both rapid eye movement sleep (REM) and NREM equally [57]; however, these are known to cause more physiological stress, as a result of motor activity or electric shock employed to keep the animals awake, and may thusly introduce potential confounding effects into experiments. All three methods raise concerns—it is almost impossible to design a “perfect control” for SD experiments [53]. To minimize the potential bias caused by a specific sleep deprivation procedure, we used two methods to cause total sleep deprivation: gentle handling (GSD) and forced locomotion facilitated by a punitive method of mild electric shock (LSD) (Fig 1A). For both the GSD and LSD treatment groups, mice were kept awake for 10 hours during their normal sleep time to ensure that the animals were sleep-deprived but were not subjected to possible interfering effects caused by extremely prolonged wakefulness. The control groups were kept undisturbed during the 10-hour treatment periods at the same time of the day. We analyzed the changes at the level of the entire brain, rather than as specific brain areas for two reasons. First, use of whole brain sampling avoids variations introduced by dissection, which can be difficult to control. Secondly, we sought to evaluate the function(s) of sleep in the entire brain. It is likely that sleep affects broad swaths of the brain, in multiple regions, as sleep deficiency is known to result in multiple types of mental impairments. including cognitive, emotional, and motor dysfunctions [1]. Clinical evidence has suggested that, instead of being driven at whole brain level, sleep might act as a fundamental self-organizing property of neuronal networks locally [58]. In addition, local sleep phenomena were also observed in rats that were awake [59]. It is quite possible that sleep is a local process that is dependent on prior activity in each neuronal network, and initiated by metabolically-driven changes [58]. Based on this theory, we hypothesized that changes in the protein levels of whole-brain samples should reflect sleep’s function as a common property of every group of neurons.

Membrane proteins such as neurotransmitter receptors, transporter, cell adhesion molecules (CAMs), and membrane enzymes are known to mediate important functions in the nervous system; membrane proteins together represent more than 60% of the targets of all modern medicinal drugs. To increase the identification rate of membrane proteins [60], total protein extracts were separated into a soluble fraction and an insoluble fraction, the latter of which was enriched for membrane proteins. Following processing according to the FASP method [39], both the soluble and the insoluble fractions were reconstituted in 4% SDS, denatured with 8M urea in filtration units, washed with ammonium bicarbonate, and digested on filter membranes with trypsin. To facilitate protein quantitation, the resulting peptides from a SD group and a matched control group were labeled with TMT-127 and TMT-126, respectively (Fig 1A). The average labeling efficiency was 99.3%. Labeled GSD or LSD peptides were mixed with the matched control peptides at a 1:1 ratio to produce the CG (Control + GSD) or CL (Control + LSD) samples for both the soluble and the insoluble fractions. To reduce complexity during the analysis, each sample was separated into ten fractions using strong cation exchange (SCX) chromatography before being analyzed using reverse-phase liquid chromatography coupled with tandem mass spectrometry (RPLC-MS/MS). Thus, four sets of data were generated for each of the three biological repeats—the soluble and insoluble fractions of the control-GSD pair (CG-S and CG-I, respectively) as well as the soluble and insoluble fractions of the control-LSD pair (CL-S and CL-I, respectively) (Fig 1A). In each sample, 7687–10844 proteins were identified at a FDR ≤ 1%.

**Fig 2:**
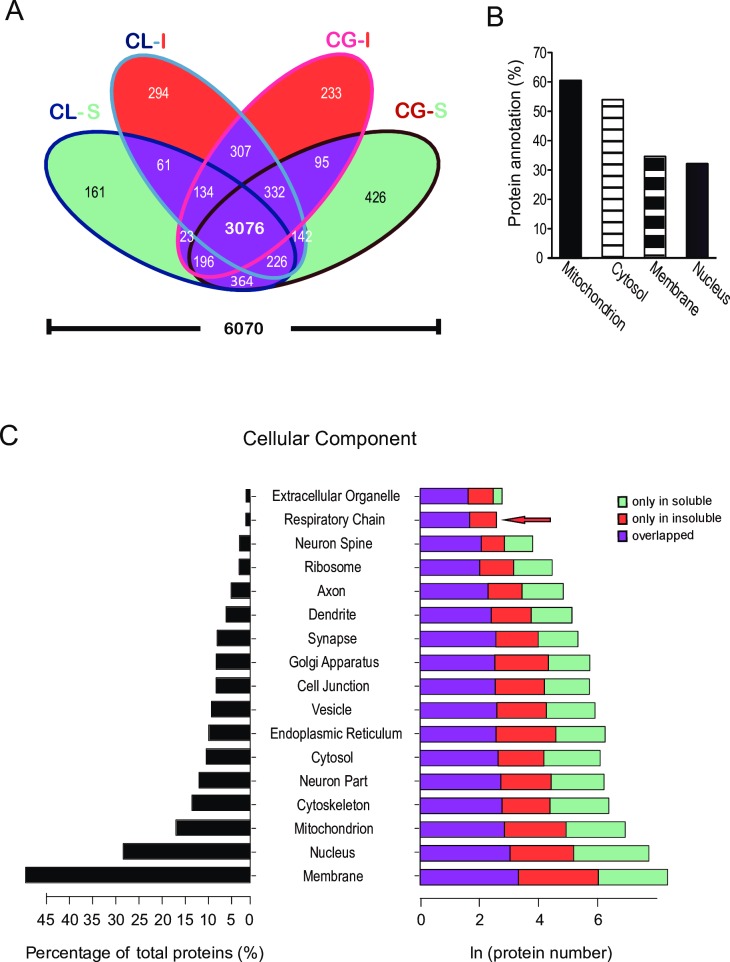
Quantified proteins are expressed in all major cellular components. (**A**) A Venn diagram showing that more than 50% of the 6070 quantified proteins (protein isoforms encoded by the same gene are merged) are shared among the soluble and insoluble fractions of both the CG and CL groups. (**B**) The percentage of quantified proteins among all annotated proteins for each GO term of the cellular components category. (**C**) For each cellular component GO term listed in the middle, the distribution (%) of these proteins among all quantified proteins is shown on the left, and the number of proteins identified and quantified only in the soluble fraction, or only in the insoluble fraction, or in both, is shown on the right. Note that no proteins of the respiratory chain were uniquely identified and quantified in the soluble fractions (red arrow).

Peptides and proteins were identified through database searching using ProLuCID [41] followed by DTASelect [45] and subsequent quantitation was carried out using Census [42] (Fig 1B). We also analyzed the data using Proteome Discoverer (PD). We quantified 21,429 and 12,862 peptides per sample using Census and PD, respectively, and the peptide quantitation results obtained using these two data analysis pipelines were in good agreement with each other (Pearson correlation coefficient Rp = 0.90 and Spearman correlation coefficient Rs = 0.87). When the Census and the PD quantitation results for the same peptide contradicted each other, the MS2 spectra of the peptide typically contained fragments of a co-eluting peptide with a similar m/z value. Therefore, these spectra were discarded from further analyses, a step that filtered out 18% of the peptides and further improved the agreement between the Census and PD quantitation results (Rp = 0.92 and Rs = 0.90). In total, we calculated relative abundance ratios for 9888 proteins, each with more than two peptides quantified (S1 Table). In the later Gene Ontology (GO) and pathway analyses, we merged the isoforms transcribed from the same gene, yielding a list of 6070 genes (S1 Fig and Fig 2A).

We assessed the reproducibility of the results at three levels: we examined the quantified protein ratios among the technical replicates, examined the overlap of quantified proteins among the biological replicates, and the examined the quantified protein ratios among the biological replicates. To assess the technical repeatability, we analyzed a CL-I sample in two technical repeats. As expected, there was good agreement between these two technical repeats (Rp = 0.90 and Rs = 0.92; S1A Fig). Also, there was extensive overlap in the proteins quantified in the three biological replicates of both the CG and the CL group (S1B Fig); the overlap between the CG and CL (three replicates merged for each) showed that—6771 proteins, encoded by 4800 genes, were quantified in both the CG group and the CL group (S1C Fig). Proteins encoded by 951 genes were found only in the soluble fractions, and proteins encoded by 834 genes were found only in the insoluble fractions (S1D Fig). Half of the gene products (3076 out of 6070) were quantified in all four groups of samples (CL-S, CL-I, CG-I, CG-S) (Fig 2A).

However, for individual proteins, the quantified ratios among biological repeats in either the CG or CL groups showed large variations (sheet B in S1 Table). Considering that we had well-matched control groups, a well-established quantitation method based on TMT labeling and high-resolution, high-mass accuracy mass spectrometry, two data analysis pipelines to validate each other, and good technical reproducibility, we suspect that a significant portion of the variation observed between these biological replicates may have to do with a multi-pathway (various inter-connected feedback regulatory cascades) and/or multi-stage responses to sleep deprivation. It is possible that fluctuations of individual proteins may converge to produce similar physiological effects. We therefore explored whether changes at the pathway level were more informative for the understanding of sleep.

### Sleep deprivation has a strong effect on mitochondrial proteins that are involved in energy metabolism and the mitochondrial unfolded protein response

To understand the subcellular consequences of sleep deprivation, we carried out GO analysis using the categories “cellular components” and “biological processes”. Of the proteins with a “cytosol” GO term in both categories, 53.8% were quantified in this study (Fig 2B); these constituted 9.5% of the total quantified proteins (Fig 2C). Of the proteins with a “membrane” GO term under the cellular component category, 34.5% were quantified in this study (Fig 2B); these constituted 48.5% of the total quantified proteins (Fig 2C). Of the proteins with a "nucleus" GO term under the cellular components category, 32.0% were quantified (Fig 2B), which accounted for 27.4% of all quantified proteins (Fig 2C). In addition, 923 mitochondrial proteins were quantified, which accounted for 60.0% of the proteins with a “mitochondrion” GO term under the cellular components category (Fig 2B). Interestingly, most proteins were typically detected in both the soluble and insoluble fractions, but those belonging to the respiratory chain were relatively more enriched in the insoluble fractions (Fig 2C).

**Fig 3:**
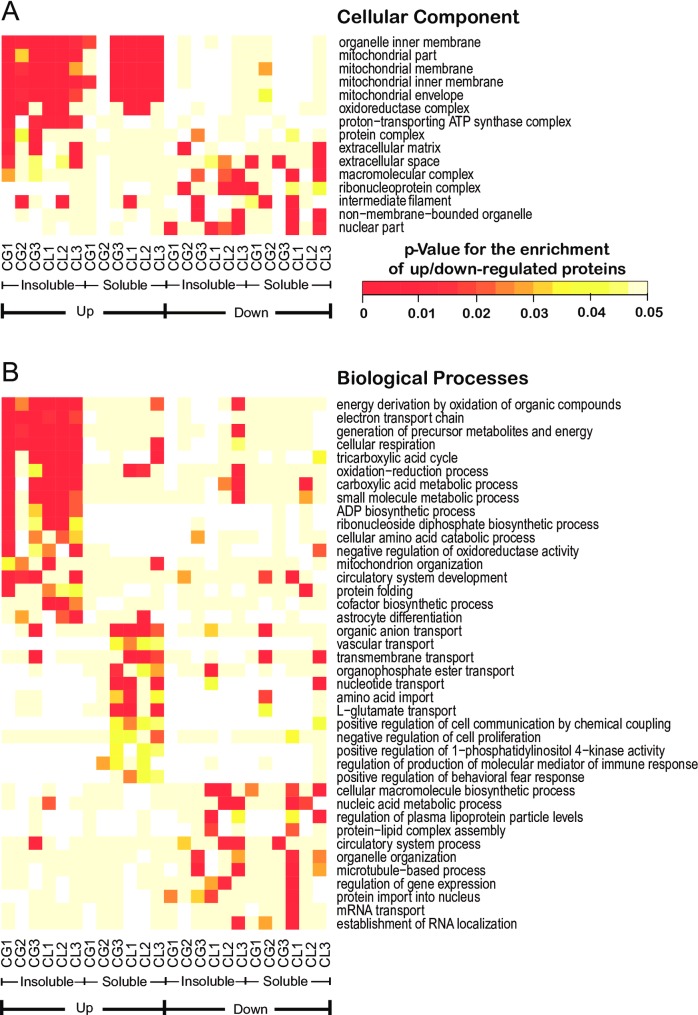
Sleep deprivation affects proteins that are associated with multiple subcellular components and biological processes, especially mitochondrial proteins that functioning in energy and small molecule metabolism. (**A**) Enrichment of GO terms in the 'cellular components' category among the up- and down-regulated proteins in the insoluble and soluble fractions of all three biological replicates from CG and CL. As shown in the heat map, the proteins up-regulated by sleep deprivation are significantly and consistently enriched for GO terms related to mitochondria, especially the mitochondrial inner membrane. (**B**) Enrichment of GO terms in the 'biological processes' category among the up- and down-regulated proteins. Up-regulated proteins from the insoluble fractions are significantly and consistently enriched for GO terms related to energy and small molecule metabolism, while those from the soluble fractions are enriched for GO terms of transport function. In contrast, proteins down-regulated by sleep deprivation were miscellaneous in their distribution, and were less consistent. The color scale of the *p* values for the significance of enrichment is the same as the scale in (A).

We calculated the deviation of each protein from the normalized interquartile range (NIQR) of all quantified proteins within individual replicates, and defined those whose ratios were over 1+2* NIQR or under 1–2*NIQR as being up or down regulated, respectively [49]. To our surprise, despite the different levels of stress caused by the GSD and LSD handling procedures, the GO analysis showed high similarity and consistency between the two groups (Fig 3). Comparison of the three biological replicates of CG-S, CL-S, CG-I, and CL-I showed that the most significantly up-regulated proteins were consistently mitochondrial proteins. Mitochondrial inner membrane proteins were particularly well-represented among the significantly up-regulated proteins. From the insoluble fractions, proteins up-regulated in the SD brains are enriched for proteins involved in energy metabolism and small molecule metabolism. From the soluble fractions, up-regulated proteins are enriched for the ones functioning in a number of transport processes including vascular transport, nucleotide transport and amino acid import (Fig 3B). The proteins down-regulated by sleep deprivation were moderately enriched for the GO terms “extracellular space”, “macromolecular complex”, and “nuclear part” (Fig 3A), but showed a relatively more diverse distribution than the up-regulated proteins. Thus, we mainly focused our further analysis on up-regulated proteins. By comparing two different sleep deprivation methods, we identified the mitochondrion as the most disturbed cellular organelle in response to prolonged wakefulness in the whole mouse brain.

**Fig 4:**
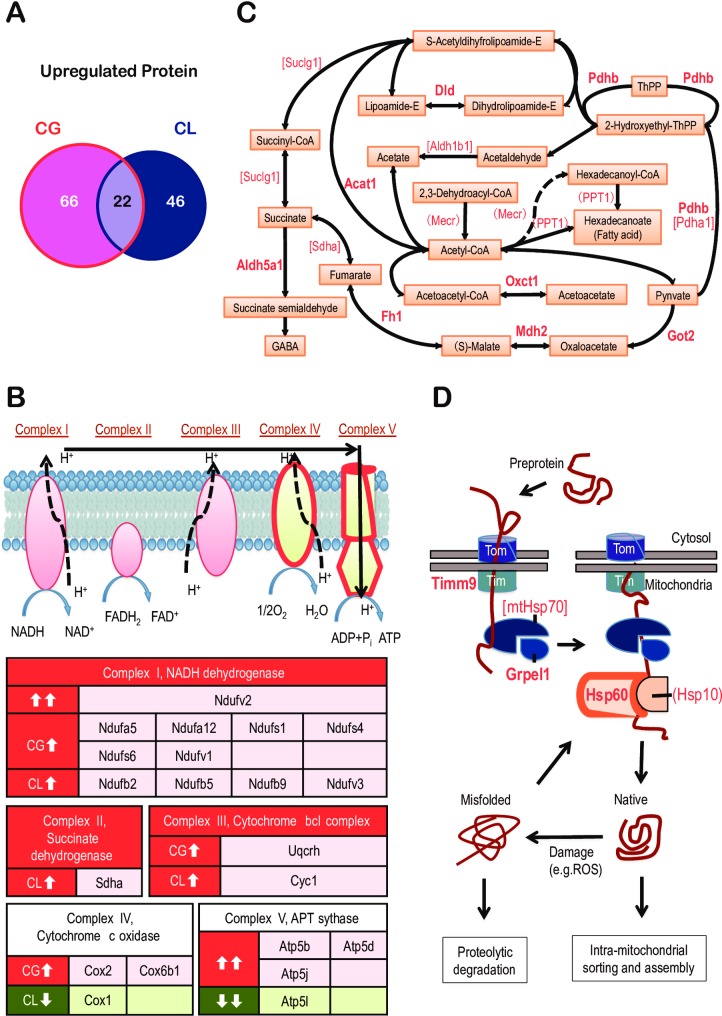
Sleep deprivation resulted in the up-regulation of proteins involved in cellular respiration. (**A**) Venn diagrams showing the distribution of significantly up-regulated proteins in the CG and CL groups. The overlap of 22 overlapped proteins is detailed in *Table 1*. (**B**) All of the complexes in the electron transport chain were influenced by sleep deprivation. Complex I-III had only up-regulated subunits, but Complex IV and V had both up- and down-regulated subunits. Dysregulated proteins are listed in the coloured table. Up-regulated proteins are in red boxes; down-regulated proteins are in green. Double arrows represent changes common to both the CG and CL sample groups. (**C**) Proteins involved in small molecule metabolism were collectively up-regulated. Metabolic compounds are framed in orange boxes, and connected with one another with solid or dashed lines, which indicate direct or indirect conversions, respectively. Enzymes that are found up-regulated from the CG or the CL groups are enclosed in parentheses or square brackets, respectively, or presented with bold font if increased in both groups. (**D**) After being synthesized in the cytosol, preproteins enter the mitochondria through the translocase of the outer membrane (TOM) followed by the translocase of the inner membrane (TIM). TIMM9 was up-regulated in both SD groups. During its transit, polypeptide can be bound and stabilized by mtHsp70 (encoded by Hspa9) with the help of Grpel1. Next, the preprotein is handed over to the complex composed of Hsp60 (encoded by Hspd1) and Hsp10 (encoded by Hspe1) and is further assisted with folding. Upon reaching their native states, mature mitochondrial proteins are sorted to their final destinations. Proteins damaged either by ROS attack or misfolding are repaired by entering refolding cycles or are degraded with the assistance of these chaperones. Chaperones that are found up-regulated from the CG or the CL groups are enclosed in parentheses or square brackets, respectively, or presented in bold font if found to be increased in both groups.

We next investigated how sleep deprivation affected the abundance of individual proteins (detailed selection method described in the “Bioinformatics and statistics” section in Materials and Methods). We found that the expression levels of 88 proteins increased significantly and consistently as a result of GSD, while 68 proteins increased significantly and consistently as a result of LSD (Fig 4A, **sheet A in S1 Table**). There was an overlap of 22 proteins whose abundance increased significantly and consistently after 10 hours of sleep loss, regardless of the handling procedure, and four decreased (Fig 4A, Table 1). Consistent with the GO enrichment analysis result, there were 20 mitochondrial proteins out of the 22 up-regulated proteins (CG, 38 of 88; CL, 31 of 68). Our analysis of the individual proteins showed that the down-regulated proteins were more diverse in their functional annotations; there was moderate enrichment in several GO terms including gene expression (Eef2, Prmt5) and macromolecule related process (Vps35) (S1 Table).

**Table 1:** Proteins showing abundance changes after sleep deprivation by both GSD and LSD.

	Gene	Protein (IPI)	Fullname	Location	Molecular Function
**Up**	**Atp5b**	IPI00468481.2	ATP synthase, H+ transporting mitochondrial F1 complex, beta subunit	mitochondrion	hydrogen ion transmembrane transporter
	**Atp5d**	IPI00453777.2	ATP synthase, H+ transporting, mitochondrial F1 complex, delta subunit	mitochondrion	hydrogen ion transmembrane transporter
	**Atp5j**	IPI00125460.1	ATP synthase, H+ transporting, mitochondrial F0 complex, subunit F	mitochondrion	hydrogen ion transmembrane transporter
	**Timm9**	IPI00125513.1	translocase of inner mitochondrial membrane 9	mitochondrion	chaperone binding
	**Grpel1**	IPI00117083.1	GrpE-like 1, mitochondrial;grpE protein homolog 1, mitochondrial	mitochondrion	chaperone binding
	**Hspd1**	IPI00308885.6	60 kDa heat shock protein, mitochondrial	mitochondrion	chaperone binding
	**Hspe1-rs1**	IPI00120045.1	heat shock protein 1 (chaperonin 10), related sequence 1	mitochondrion	chaperone binding
	**Mdh2**	IPI00323592.2	malate dehydrogenase 2	mitochondrion	oxidoreductase activity
	**Ndufv2**	IPI00169925.2	NADH dehydrogenase (ubiquinone) flavoprotein 2	mitochondrion	oxidoreductase activity
	**Pdhb**	IPI00132042.1	pyruvate dehydrogenase (lipoamide) beta	mitochondrion	oxidoreductase activity
	**Aldh5a1**	IPI00273164.1	aldhehyde dehydrogenase family 5, subfamily A1	mitochondrion	oxidoreductase activity
	**Dld**	IPI00874456.1	dihydrolipoamide dehydrogenase	mitochondrion	oxidoreductase activity
	**Prdx5**	IPI00759999.1	peroxiredoxin 5	mitochondrion;	oxidoreductase activity
		IPI00129517.1		membrane bounded vesicle	
	**Oxct1**	IPI00132653.1	3-oxoacid CoA transferase 1	mitochondrion	transferase activity
	**Acot13**	IPI00132958.1	acyl-CoA thioesterase 13	mitochondrion	transferase activity
	**Got2**	IPI00117312.1	glutamate oxaloacetate transaminase 2	mitochondrion	transaminase activity
	**Acat1**	IPI00154054.1	acetyl-Coenzyme A acetyltransferase 1	mitochondrion	hydrolase activity
	**Fh1**	IPI00129928.2	fumarate hydratase 1	mitochondrion;	hydrolase activity
				membrane bounded vesicle	
	**Nipsnap1**	IPI00515151.3	protein NipSnap homolog 1	mitochondrion;	neurotransmitter binding
		IPI00115824.1		synapse membrane	
	**Cav1**	IPI00117829.1	caveolin 1	mitochondrion;	kinase binding;
				membrane bounded vesicle	peptidase activator
	**Lamb2**	IPI00109612.2	laminin subunit beta-2 precursor	extracellular matrix;	integrin binding
			laminin subunit beta-2	membrane bounded vesicle	
	**Col1a2**	IPI00988109.1	collagen, type I, alpha 2	extracellular matrix;	SMAD binding;
				membrane bounded vesicle	calcium ion binding
**Down**	**Atp5l**	IPI00133342.1	ATP synthase, H+ transporting, mitochondrial F0 complex, subunit G	mitochondrion	hydrogen ion transmembrane transporter
	**Eef2**	IPI00466069.3	eukaryotic translation elongation factor 2	cytosol; polysome;	protein kinase binding;
				membrane bounded vesicle	GTPase activity
	**Prmt5**	IPI00831158.1	protein arginine N-methyltransferase 5	cytosol; nucleus	PRMT activity; HMT activity
	**Vps35**	IPI00111181.1	vacuolar protein sorting 35	lysosomal membrane;	protein binding
				retromer complex	

PRMT, protein-arginine omega-N asymmetric methyltransferase; HMT, Histone methyltransferases

Notably, proteins in the respiratory chain were strongly disturbed by SD. In both the GSD and LSD brains, four mitochondrial respiratory chain proteins were significantly up-regulated: one from Complex1 (Ndufv2), and three from mitochondrial ATP synthase (Atp5b, Atp5d, and Atp5j). There were nine additional respiratory chain proteins (Ndufa5, Ndufa12, Ndufs1, Ndufs4, Ndufs6, Ndufv1, COX2, Cox6b1, and Uqcrh) up-regulated in the CG group, and seven (Ndufb2, Ndufb5, Ndufb9, Ndufv3, Sdha, and Cyc1) up-regulated in the CL group (Fig 4B).

These changes in the abundance of proteins that function in cell respiration were accompanied by other changes in metabolism of small molecules (Fig 3B). As shown in Fig 4C, the TCA cycle, pyruvate metabolism, and branched-chain amino acid (Val, Leu and Ile) degradation were strongly affected by sleep deprivation (Dld, Pdhb, Aldh5a1, Fh1, Mdh2, Acat1, Oxct1, and Got2).

Another major outcome of sleep deprivation is the overexpression of mitochondrial chaperone proteins. The mtHsp70 (encoded by *Hspa9*) and Hsp60 (encoded by *Hspd1*) family members have been recognized as major players that facilitate the folding of nuclear-encoded proteins when they are imported into mitochondria [61,62] (Fig 4D). Hsp60, Hsp10, and mtHsp70 are organelle-specific biomarkers that are considered to reflect mitochondria stress [63]. In this study, sleep deprivation significantly and consistently caused up-regulation of Hsp60 and Grpel1, known regulators of mtHsp70 (Table 1). Hsp10 (encoded by *Hspe1*) in the CG group, and mtHsp70 in CL group, were also found to be up-regulated (S1 Table). The levels of two hypothetical chaperone proteins, Hspe1-rs1 (chaperonin 10 related sequence 1) and Timm9 [64], also increased following sleep deprivation. These results suggested that sleep deprivation triggered mitochondrial stress and induced the expression of proteins in the mitochondrial chaperone network (Fig 4D).

To validate the MS quantitation results using an orthogonal method, we analyzed whole-brain lysates via western blotting to measure the mitochondrial proteins levels both before and after sleep deprivation. Among the 12 antibodies we tested, the Hsp60protein 2.ss bundance in sleep-deprived mice. ent increaseof what'd uld much rather to giveel studies. formation. weak. HS and Sod2 (superoxide dismutase 2, mitochondrial) antibodies showed good signals. As shown in S2B Fig, Hsp60 (Hspd1)protein 2.ss bundance in sleep-deprived mice. ent increaseof what'd uld much rather to giveel studies. formation. weak. HS was consistently up-regulated, and Sod2 levels were variable among different biological replicates; these findings both reinforced our MS results. Considering the controversial role of Sod2 in sleep loss suggested by other groups [65–69], the variation in the Sod2 levels among different individuals is perhaps not surprising. In addition to mitochondrial proteins, there was a notable increase of proteins related to the extracellular matrix and to membrane bound vesicles, including caveolin (Cav1), laminin (Lamb2, Lama5, Lamc1), and collagen (Col1a2, Col12a1) (Table 1 and S1 Table).

### Sleep deprivation results in significant changes in the abundance of proteins related to homeostasis, mental function, and neurodegenerative diseases

**Fig 5:**
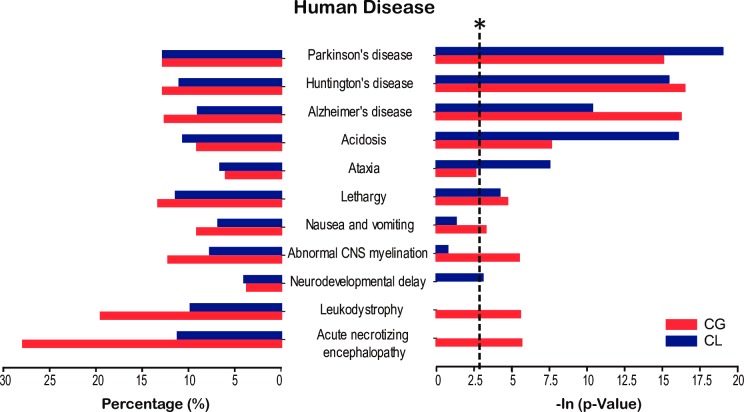
Proteins up-regulated by sleep deprivation are linked to neurodegeneration diseases. According to the annotations of the KEGG Disease and the Human Phenotype Ontology databases, the 140 up-regulated proteins in the CG or CL groups, or both, were enriched for proteins associated with human diseases. The bars show the percentage (left) and–ln(*p* value) (right) of the enriched annotation categories (*, p<0.05, -ln(p value) >3). Up-regulated proteins are significantly enriched in Parkinson’s disease, Huntington’s disease, and Alzheimer’s disease.

To further explore the potential pathological relevance of sleep deprivation, we checked whether any of the proteins with higher abundance after ten hours of sleep deprivation were known to be associated with human diseases. We used tools available with the Human Phenotype Ontology [51] and KEGG Disease databases. Consistent with the reported behavioral effects of sleep deprivation [2,70], we found that some of the up-regulated gene products observed in our study were significantly clustered in gene groups associated with ataxia and lethargy (Fig 5). Proteins related to acidosis or abnormalities in metabolism/homeostasis were also enriched. To our surprise, the most enriched pathways in both GSD and LSD mice were found to be those known to mediate neurodegenerative diseases, including Parkinson’s disease, Huntington’s disease, and Alzheimer’s disease. These results suggested a possible link between sleep deprivation and neurodegeneration.

## Discussion

Here, we used advanced quantitative proteomics technologies to examine changes in the proteome of the entire mouse brain following sleep deprivation. We quantified nearly 10,000 proteins encoded by over 6000 genes, covering all major cellular components. We found that sleep deprivation had the strongest effect on the abundance of proteins related to mitochondrial stress responses and energy metabolism. We also found that proteome changes following SD seem to indicate a link between SD and neurodegenerative diseases.

To ensure that our results were as rigorous as possible, we used two different sleep deprivation treatment protocols (GSD and LSD). Each of these experiments tested three biological repeats. We also used advanced proteomics technologies and used two parallel software pipelines for data analysis. Although the punitive aspect of the LSD protocol may have induced undesired stress and may have activated the limbic system, it is important to note that the signaling pathways of the GSD and LSD that were up-regulated were largely in agreement. The LSD mice showed mild down-regulation in GO term “nucleic acid metabolic process” and “regulation of plasma lipoprotein particle levels” compared with the GSD mice (Fig 3B), which may have resulted from additional stress or perhaps from the increased amount of exercise.

Our knowledge about which signaling pathways are affected by sleep deprivation has been garnered mainly from transcriptomic studies. Given that translational and post-translational regulation mechanisms are known to exist widely, the examination of changes in protein abundance provides direct and indispensable information about the functional consequences of sleep deprivation. Previous microarray studies have suggested that mitochondria exhibit transcriptional responses following three hours of sleep deprivation treatment [13]. This is consistent with the findings of Dworak *et al*., who reported that ATP levels surge in wake-active brain regions, and this surge can be abolished by preventing sleep for three or six hours [71]. However, changes in the mitochondrial transcriptome are transient; they are not detected after eight hours [13]. It has been proposed that the energy demand in the cerebral cortex first increases and then decreases during prolonged wakefulness [72]. However, western blot analysis of the subunits of enzymes in the electron transport chain indicated that cortex COXI and COXIV protein levels increased significantly after both 3 and 12 h of sleep deprivation [73]. The present quantitative proteomics study revealed that this elevation in the levels of brain mitochondrial proteins remains prominent after 10 h of sleep deprivation. Viewed together, the results of these various studies suggest that mitochondrial functions are affected continuously throughout periods of prolonged wakefulness.

The conservation of energy and the restoration of macromolecules may be two sides of the same coin in the functions of sleep. When chemical equilibrium deviates from homeostasis during prolonged wakefulness, cellular stress may accumulate and activate defense responses at some point [74]. Several microarray studies have found that more than six hours of sleep deprivation causes the overexpression of genes that encode proteins involved in the endoplasmic reticulum unfolded protein response (ER-UPR) as well as the mitochondrial unfolded protein response (MT-UPR) in the brain cortex [17,75,76]. In the present study, we observed MT-UPR (Hsp60, Hsp10, Hspe1-rs1, mtHSP70, Gprel1) but did not observe ER-UPR. Hsp60 has been suggested to play a role in the cytosol [75,77]. Interestingly, our data showed that Hsp60 was up-regulated in the insoluble fraction but not in the soluble fraction (S2B Fig, S1 Table). Considering that the importation of a large amount of mitochondrial proteins relies on the electrochemical potential of the inner mitochondrial membrane [78,79], we suggest that MT-UPR results from the impairment of the respiratory chain that occurs during prolonged wakefulness and, further, up-regulation of the ETC proteins is therefore a compensatory response undertaken to maintain energy metabolism homeostasis.

Our results suggest a connection between sleep loss, mitochondria stress responses, and neurodegeneration. A series of recent reports have highlighted the role of mitochondria in neurodegenerative diseases [80–82]. A specific defect of Complex I activity has been found not only in the substantia nigra, but also in other brain areas of patients with Parkinson’s disease [83,84]. In Alzheimer's disease, oxidative stress and mitochondrial dysfunction occur early, and often progress with severity[85,86]. We hypothesize that cellular stress induced by short-term or acute sleep deprivation originates from impairment of mitochondrial ETC complexes; this then disrupts energy metabolism and triggers MT-UPR. We speculate that long-term or chronic sleep deprivation may cause irreversible mitochondrial dysfunction that results in the production of reactive oxygen species (ROS) and increases calcium leakage, accelerating disease progression.

Although there is as yet no consensus about the function of sleep, previous studies of sleep and sleep deprivation have suggested that the functions of sleep include a role in learning and synaptic plasticity, energy conservation, and physiological recovery at the cellular, neural network, and endocrine system levels [87]. Accumulating evidence lends support to the hypothesis that sleep is critical for synaptic homeostasis in the cortex region [59,88–90]. However, at the whole-brain level, we did not observe consistent changes of proteins related to synaptic plasticity, even though we identified and quantified numerous synaptic proteins and neurotransmitter receptors. It is possible that changes in the abundance of synaptic proteins may be restricted to particular brain areas, and these changes could have been masked by our whole-brain sampling experimental design. The use of whole brains as the study subject was thought to be conductive to the identification of global changes, as we treated various brain cells (including neurons and glial cells) as equal. However, in order to understand the consequences of sleep deprivation more thoroughly, future studies should analyze wake-active and sleep-active brain regions separately. Further, studies that examine changes in protein levels in specific cell types in these regions may be needed to provide the data to definitively characterize the effects of sleep deprivation at the molecular level.

## Supporting Information

S1 Fig Quality control and confirmation of the quantitative mass spectrometry method. (A) Scatter plot of the correlation of the control group (TMT^126^) versus the GSD group (TMT^127^) for protein expression ratios determined by two technical replicates. Data were derived from the ICL group. The two technical repeats were very similar and thus, we did not test technical repeats in other groups. (B) Venn diagrams depicting the overlapping and unique proteins quantified in three biological replicates of the CG and CL groups. (C) Venn diagrams depicting the overlapping and unique proteins quantified in the CG and CL groups. Left panel presents the number of unique proteins. Right panel shows the number of protein-coding genes. We identified and quantified nearly ten thousand protein isoforms that are encoded by ~6000 genes, a majority of these proteins were present in both groups. (D) Venn diagrams showing the overlapping and unique proteins quantified in the soluble and insoluble fractions. Left panel presents the number of unique proteins. Right panel shows the number of protein-coding genes.
(TIF)

S2 Fig Down-regulated proteins in GSD and LSD mice and upregulation of Hsp60. (A) Venn diagrams show the distribution of significantly down-regulated proteins in the CG and CL groups. The overlap of 4 proteins is detailed in Table 1. (B) Western blot (WB) verification of selected mitochondria proteins. Whole-brain lysates were analyzed by WB with antibodies against Hsp60 and Sod2. In the MS analysis, Hsp60, but not Sod2, was found to be up-regulated in sleep-deprived brains. Relative expression ratios in SD groups compared with the control groups were obtained after normalization to total proteins. The Coomassie blue staining was shown at the bottom. The MS quantification results were also listed.
(TIF)

S1 Table sheetA: List of all differentially expressed proteins after sleep deprivation. sheetB: List of all the quantified proteins.
(XLSX)
