# Supplementary figures and images for: Quantitative Proteomics of Sleep-Deprived Mouse Brains Reveals Global Changes in Mitochondrial Proteins
Source: PLoS One. 2016 Sep 29;11(9):e0163500. doi: 10.1371/journal.pone.0163500 (PMC5042483; doi:10.1371/journal.pone.0163500)

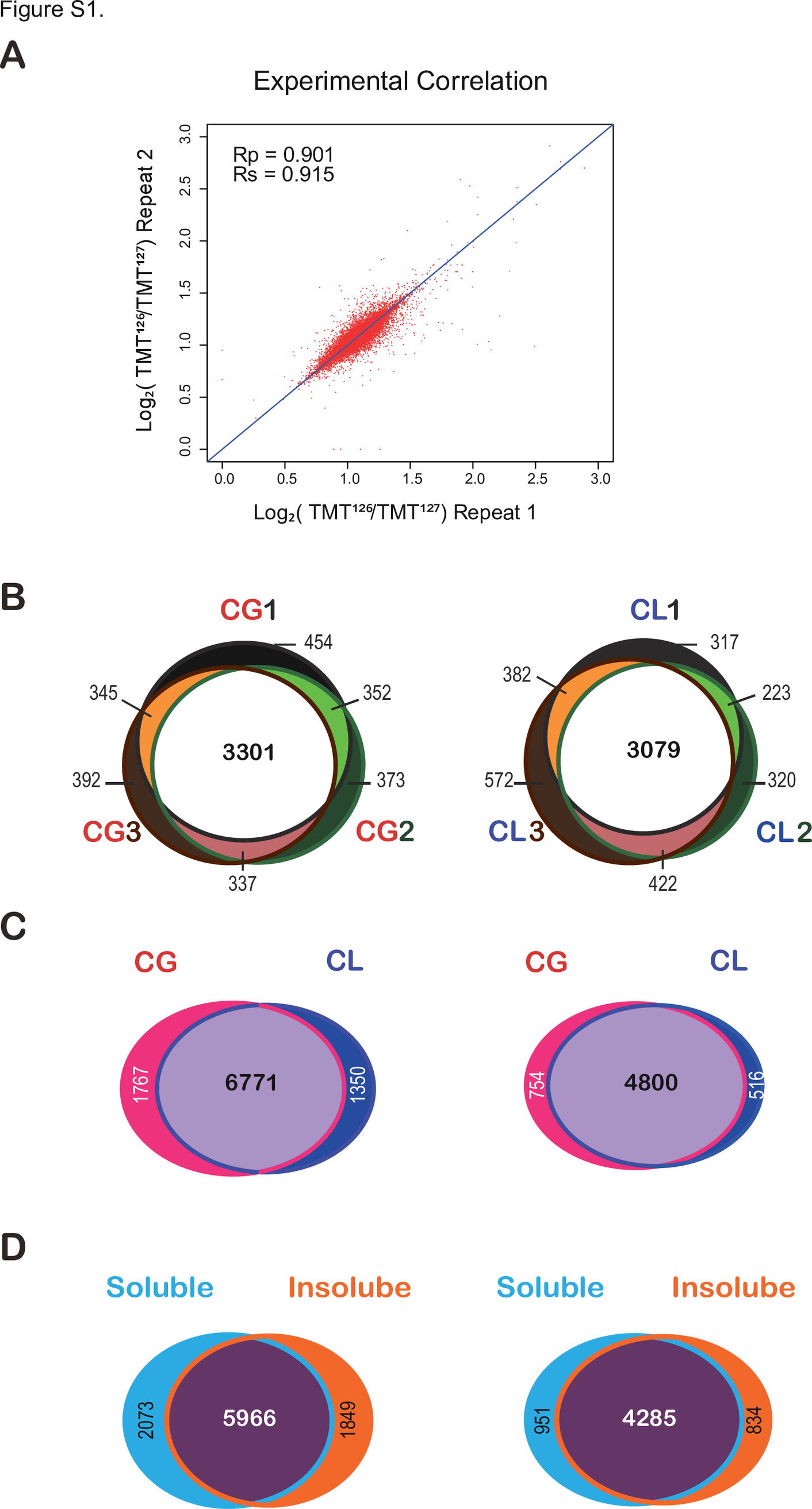

Supplement: S1 Fig — (A) Scatter plot of the correlation of the control group (TMT126) versus the GSD group (TMT127) for protein expression ratios determined by two technical replicates. Data were derived from the ICL group. The two technical repeats were very similar and thus, we did not test technical repeats in other groups. (B) Venn diagrams depicting the overlapping and unique proteins quantified in three biological replicates of the CG and CL groups. (C) Venn diagrams depicting the overlapping and unique proteins quantified in the CG and CL groups. Left panel presents the number of unique proteins. Right panel shows the number of protein-coding genes. We identified and quantified nearly ten thousand protein isoforms that are encoded by ~6000 genes, a majority of these proteins were present in both groups. (D) Venn diagrams showing the overlapping and unique proteins quantified in the soluble and insoluble fractions. Left panel presents the number of unique proteins. Right panel shows the number of protein-coding genes. (TIF) [file pone.0163500.s001.tif]

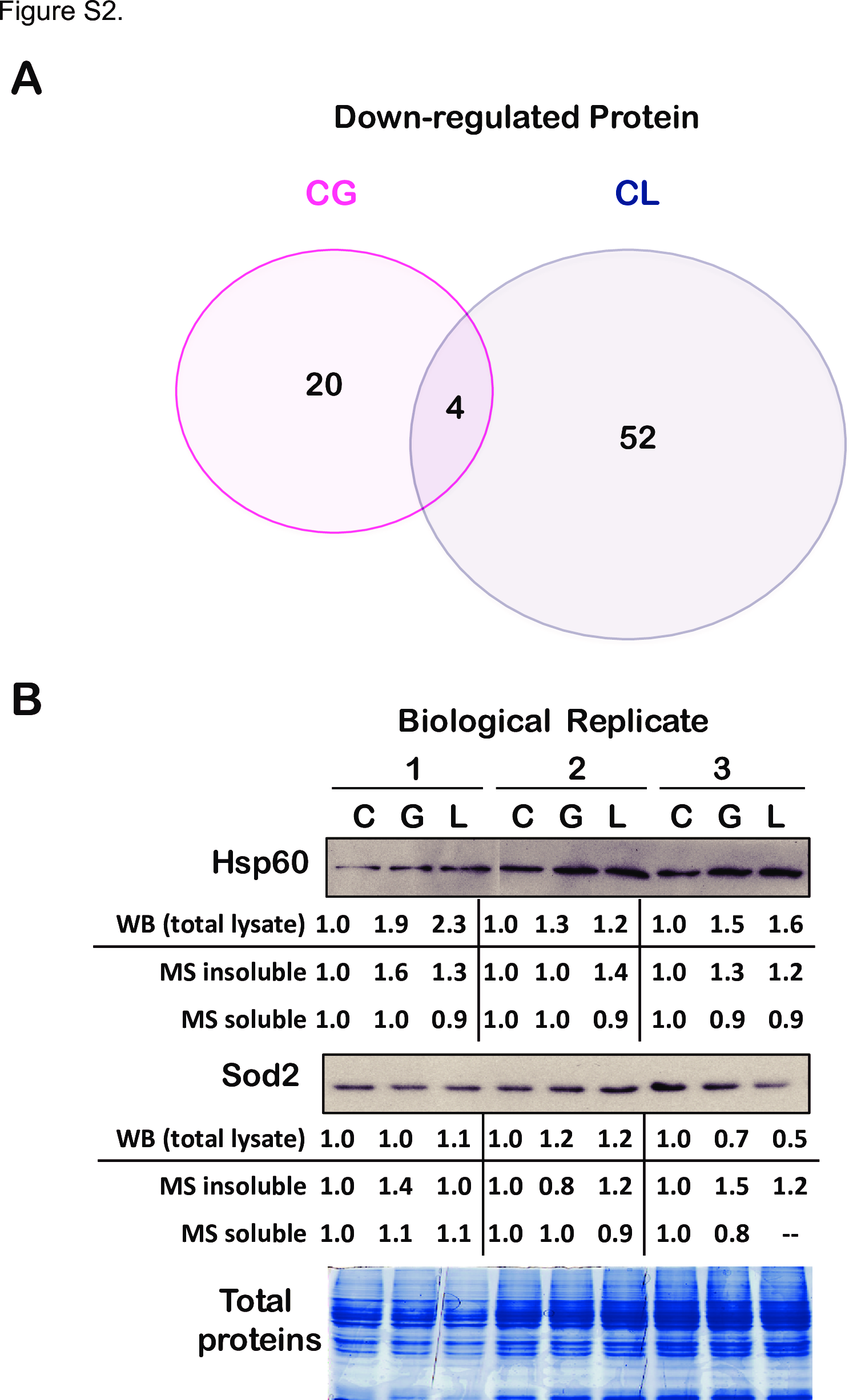

Supplement: S2 Fig — (A) Venn diagrams show the distribution of significantly down-regulated proteins in the CG and CL groups. The overlap of 4 proteins is detailed in Table 1. (B) Western blot (WB) verification of selected mitochondria proteins. Whole-brain lysates were analyzed by WB with antibodies against Hsp60 and Sod2. In the MS analysis, Hsp60, but not Sod2, was found to be up-regulated in sleep-deprived brains. Relative expression ratios in SD groups compared with the control groups were obtained after normalization to total proteins. The Coomassie blue staining was shown at the bottom. The MS quantification results were also listed. (TIF) [file pone.0163500.s002.tif]
